# Supplementary material for: Optimization of Fermentation Conditions and Metabolite Profiling of Grape Juice Fermented with Lactic Acid Bacteria for Improved Flavor and Bioactivity
Source: Foods. 2023 Jun 18;12(12):2407. doi: 10.3390/foods12122407 (PMC10297718; doi:10.3390/foods12122407)
Supplement: Supplementary file 1 [file foods-12-02407-s001.zip › foods-2425419-supplementary.pdf]

Supplementary Information to

Optimization of Fermentation Conditions and Metabolite Profiling of Grape Juice Fermented with Lactic Acid Bacteria for Improved Flavor and Bioactivity

Part 1:Determination of the strains function and optimizing the fermentation conditions (Figure S1-S4; Table S1-S5)

Part 2: Analyses of metabolites profiles during the multi-strain fermentation stages (Figure S5-S7; Table S6-S8)

Part 3: Analyses of VOCs profiles (Figure S8; Table S8-S10)

Part 1:Determination of the strains function and optimizing the fermentation conditions (Figure S1-S4; Table S1-table S5)

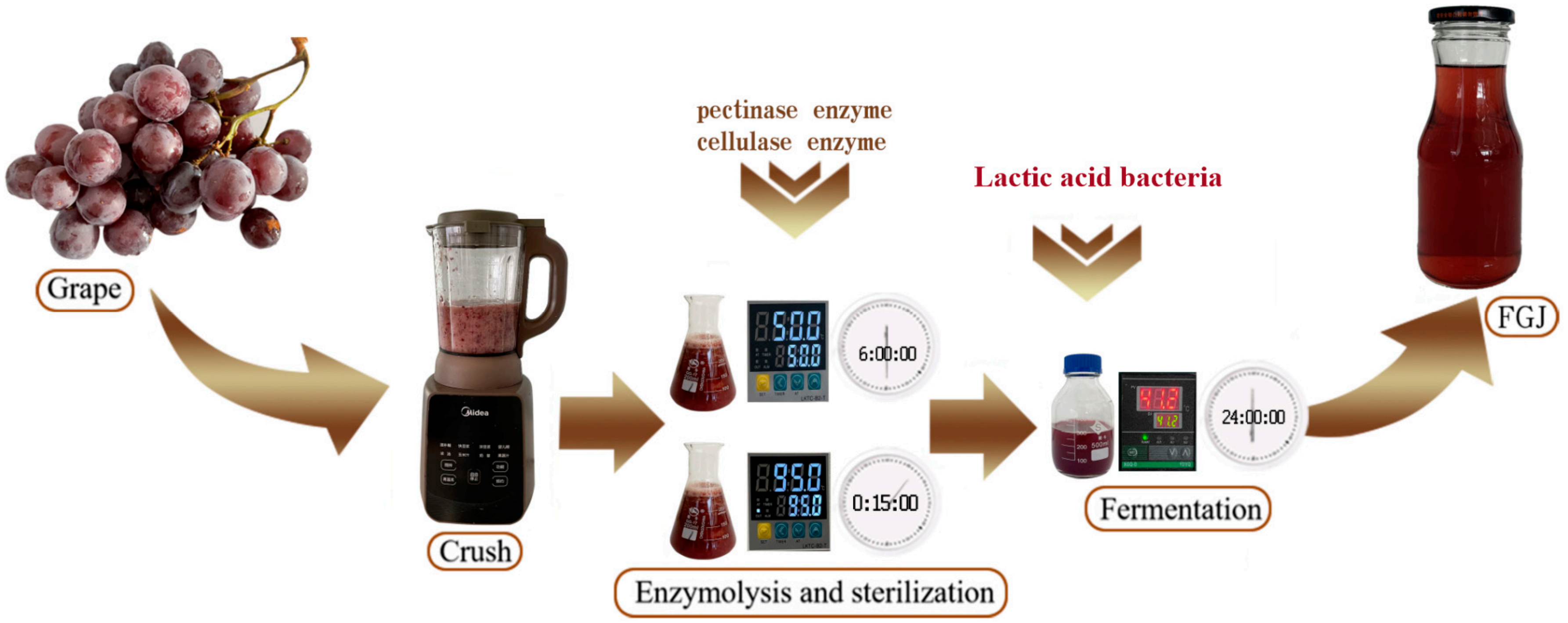

Figure S1 The diagram of process flow of product FGJ

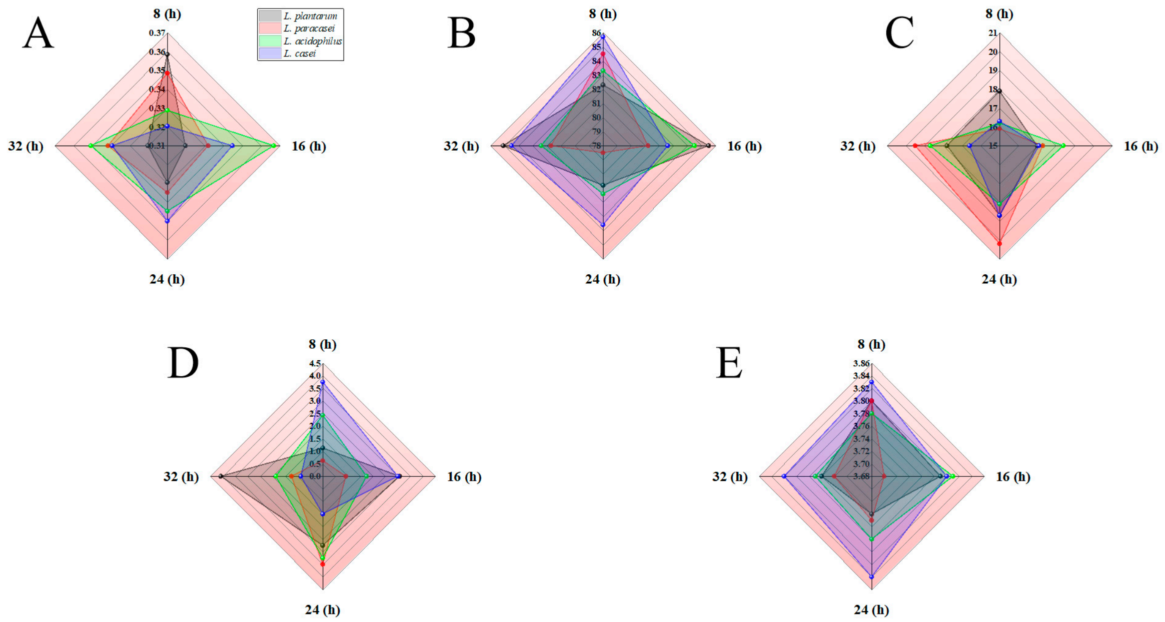

Figure S2 Radar map analysis in TPC (A), clarification (B), TSS (C), LAB density (D), and pH (E) of FGJ during different single-strain (*L. plantarum, L. acidophilus, L. casei, L. paracasei*) fermentation stages

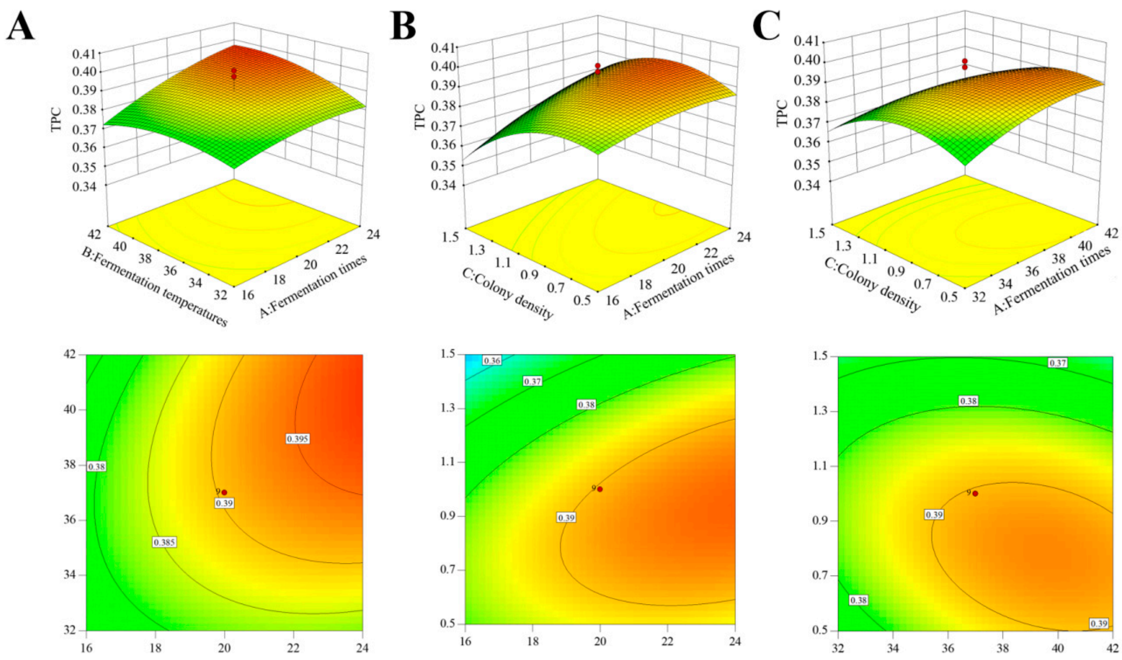

Figure S3 Three-dimensional (3D) response surfaces of different factors (A:fermentation time; B:fermentation temperature; C:Colony density) on the TPC of FGJ

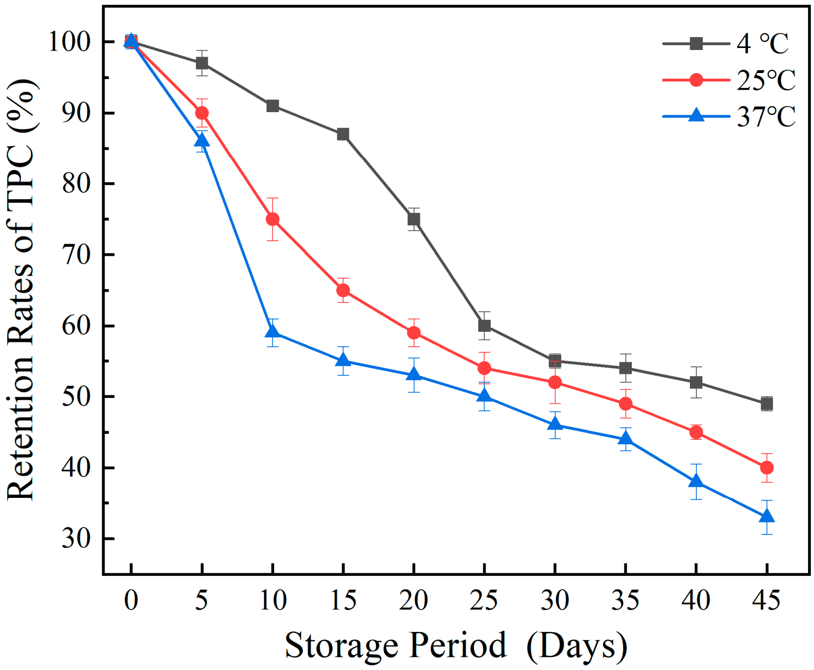

Figure S4 Changes in retention rate of TPC during accelerated storage period

Table S1 Uniform design factor level table

Factor	*L. plantarum* X1/%	*L.acidophilus* X2/%	*L. casei* X3/%	*L. paracasei* X4/%
1	0.64	0.61	0.55	0.43
2	0.65	0.65	0.58	0.46
3	0.67	0.62	0.56	0.47
4	0.63	0.66	0.52	0.48
5	0.66	0.64	0.51	0.41
6	0.62	0.67	0.57	0.42
7	0.68	0.68	0.54	0.44
8	0.61	0.63	0.53	0.45

Table S2 Results of the uniform design experiment

Factor	*L. plantarum* X1/%	*L.acidophilus* X2/%	*L. casei* X3/%	*L. paracasei* X4/%	TPC (mg GAE/mL)
1	0.64	0.61	0.55	0.43	0.341
2	0.65	0.65	0.58	0.46	0.364
3	0.67	0.62	0.56	0.47	0.355
4	0.63	0.66	0.52	0.48	0.365
5	0.66	0.64	0.51	0.41	0.351
6	0.62	0.67	0.57	0.42	0.362
7	0.68	0.68	0.54	0.44	0.379
8	0.61	0.63	0.53	0.45	0.344

Table S3 The level table of response surface factors

Level	Factors		
	A fermentation time (h)	B fermentation temperature (℃)	C LAB density (10^7CFU/mL)
-1.68	13.28	28.6	0.16
-1	16	32	0.5
0	20	37	1
1	24	42	1.5
1.68	26.72	45.4	1.84

Table S4 Experimental results of multi-strain fermentation by response surface design

Number	A<br>fermentation time (h)	B<br>fermentation temperature (°C)	C<br>LAB density (10^7 CFU/mL)	TPC<br>(mg GAE/mL)
1	16	32	1.5	0.348
2	26.73	37	1	0.392
3	24	32	0.5	0.367
4	16	32	0.5	0.373
5	20	37	1	0.386
6	20	37	1	0.385
7	16	42	1.5	0.345
8	20	37	1	0.389
9	20	45.41	1	0.377
10	24	32	1.5	0.373
11	20	37	1.84	0.343
12	24	42	0.5	0.399
13	16	42	0.5	0.378
14	20	37	1	0.387
15	20	37	1	0.388
16	13.27	37	1	0.370
17	20	28.59	1	0.374
18	20	37	0.16	0.370
19	24	42	1.5	0.377
20	20	37	1	0.401
21	20	37	1	0.398
22	20	37	1	0.390
23	20	37	1	0.390

Table S5 Analysis of variance and significance test for the response values regression model

Source of variance	Quadratic sum	Variance	Mean square	F Number	*p* Number	significance
Model	0.00544	9	0.00060	21.65	< 0.0001	**
A-Fermentation time	0.00087	1	0.00087	31.19	< 0.0001	**
B-Fermentation	0.00014	1	0.00014	4.86	0.0460	*
C-temperature	0.00104	1	0.00104	37.43	< 0.0001	**
AB	0.00014	1	0.00014	5.18	0.0404	*
AC	0.00022	1	0.00022	7.90	0.0147	*
BC	0.00016	1	0.00016	5.81	0.0315	*
A2	0.00017	1	0.00017	6.21	0.0270	*
B2	0.00044	1	0.00044	15.68	0.0016	**
C2	0.00227	1	0.00227	81.53	< 0.0001	**
Residual	0.00036	13	0.00003			
Unplanned item	0.00012	5	0.00002	0.84	0.5594	Not significant
Error term	0.00024	8	0.00003			
Total	0.00580	22				
R^2=0.9386	R^2Adj=0.8960					

Part 2: Analyses of metabolites profiles during the multi-strain fermentation stages (Figure S5-S7; Table S6-S8)

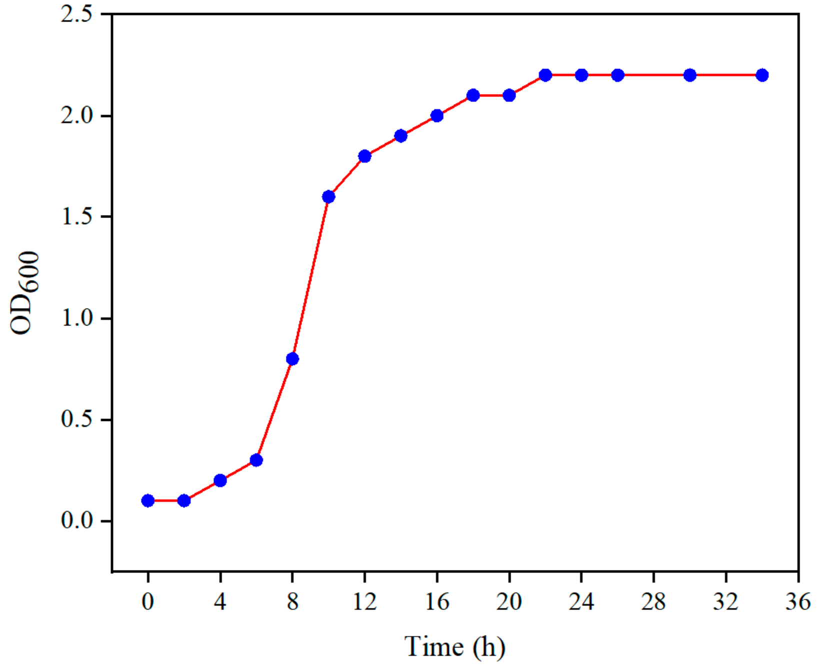

Figure S5 Growth curve analysis of multi-strain during different incubate stages

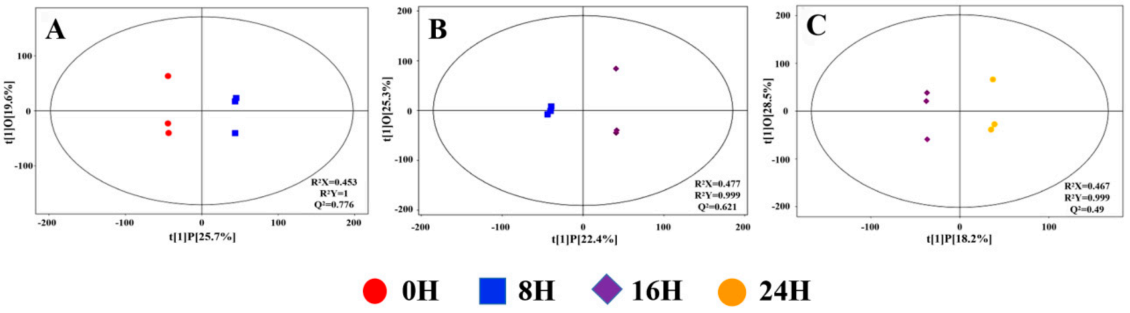

Figure S6 OPLS-DA score plots analysis of FGJ during different multi-strain fermentation stages (A: 0-8h; B: 8-16h; C:16-24h)

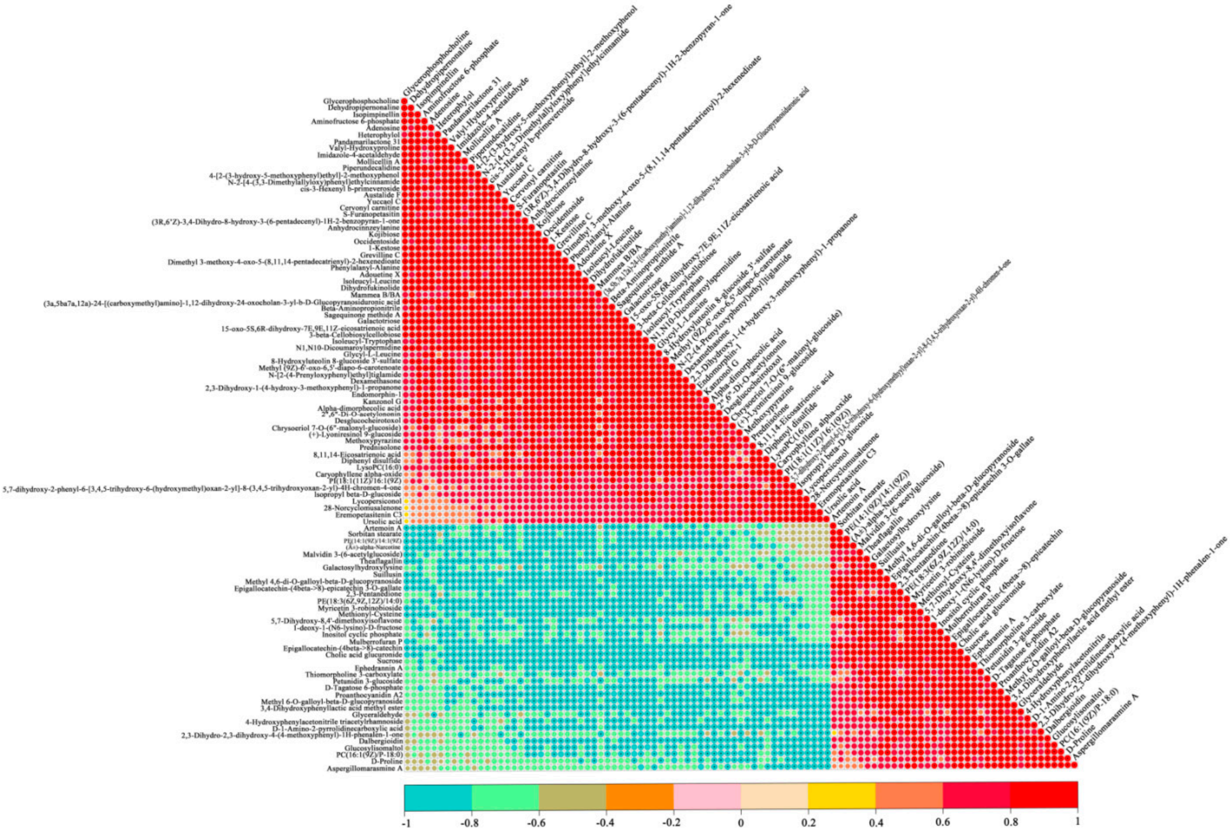

Figure S7 Correlation maps of metabolites in FGJ during primary fermentation stage

Table S6 Differential metabolites in FGJ during the three different multi-strain fermentation stages

NO	Primary fermentation stage (0-8h)	Post fermentation stage (8-16h)	Final fermentation stage (16-24h)
1	Dexamethasone	H-LEU-VAL-OH	Phytosphingosine
2	2,3-Dihydro-2,3-dihydroxy-4-(4-methoxyphenyl)-1H-phenalen-1-one	Cortisone	Protoanemonin
3	D-Proline	xi-3-(4-Isopropylphenyl)-2-methylpropanal	1-Kestose
4	Beta-Aminopropionitrile	Isoleucyl-Leucine	Tridemorph
5	Yuccaol C	Dictyoquinazol C	Theaflagallin
6	1-Kestose	LysoPE(0:0/14:0)	Rubraflavone C
7	Theaflagallin	5,7-Dihydroxy-8,4'-dimethoxy isoflavone	Palmitoylethanolamide
8	Adenosine	Diospyrin	5-Decanoyl-2-nonylpyridine
9	Occidentoside	Palmitoylethanolamide	Methyl 6-O-galloyl-beta-D-glucopyranoside
10	Petunidin 3-glucoside	Phenylalanyl-Arginine	Pyrophaeophorbide a
11	Isoleucyl-Leucine	1-(5Z,8Z,11Z,14Z-eicosatetraenoyl)-sn-glycero-3-phosphate	Tetraacetylethylenediamine
12	15-oxo-5S,6R-dihydroxy-7E,9E,11Z-eicosatrienoic acid	Artemoin A	4,4-Dimethoxy-2-butanone
13	1-deoxy-1-(N6-lysino)-D-fructose	PE(20:0/18:1(9Z))	Bismahanine
14	5,7-Dihydroxy-8,4'-dimethoxyisoflavone	Romucosine A	Polyoxyethylene (600) monoricinoleate
15	Suillusin	Bismahanine	Palmitoleoyl Ethanolamide
16	Mammea B/BA	PE(P-18:1(11Z)/22:5(4Z,7Z,10Z,13Z,16Z))	Isoleucyl-Phenylalanine
17	Phenylalanyl-Alanine	3,4-Dihydroxyphenyllactic acid methyl ester	Bisphenol A
18	Kojibiose	Ursolic acid	8-Butanoylneosolaniol

19	Heterophylol	Dihydrofukinolide	Linamarin

Table S6 Differential metabolites in FGJ during the three different multi-strain fermentation stages

NO	Primary fermentation stage (0-8h)	Post fermentation stage (8-16h)	Final fermentation stage (16-24h)
20	Imidazole-4-acetaldehyde	Prenyl caproate	Annocherin A
21	Aspergillomarasmine A	Aldosterone 18-glucuronide	Methyl 3-(2,3-dihydroxy-3-methylbutyl)-4-hydroxybenzoate
22	Caryophyllene alpha-oxide	(-)-Epigallocatechin 3-(4-methyl-gallate)	Serylisoleucine
23	2'',6''-Di-O-acetylononin	Glycyl-L-Leucine	LysoPC(20:4(5Z,8Z,11Z,14Z))
24	Methyl 6-O-galloyl-beta-D-glucopyranoside	Leucyl-Lysine	6-Methyladenine
25	Methionyl-Cysteine	Leucyl-Gamma-glutamate	4-p-Coumaroyl-1,5-quinolactone
26	Glycerophosphocholine	Dimethyl 3-methoxy-4-oxo-5-(8,11,14-pentadecatrienyl)-2-hexenedioate	D-Galactopyranosyl-(1->3)-D-galactopyranosyl-(1->3)-L-arabinose
27	N1,N10-Dicoumaroylspermidine	Trifluoromethanesulfonic acid	Lycoperoside D
28	Artemoin A	Solanocardinol	Macrocarpal I
29	Kanzonol G	PE(22:4(7Z,10Z,13Z,16Z)/18:1(11Z))	Pipericine
30	Grevilline C	Leucyl-Methionine	Malvidin 3-sophoroside 5-glucoside
31	PI(18:1(11Z)/16:1(9Z))	Linamarin	Aspartyl-Leucine
32	cis-3-Hexenyl b-primeveroside	Anhydrocinnzeylanine	(2R*,3R*)-1,2,3-Butanetriol
33	Myricetin 3-robinobioside	Thiomorpholine 3-carboxylate	3'-Glucosyl-2',4',6'-trihydroxyacetophenone
34	Isopropyl beta-D-glucoside	(R) 2,3-Dihydroxy-3-methylvalerate	Edulinine
35	PC(16:1(9Z)/P-18:0)	Ethoxyquin	PA(18:0/20:4(5Z,8Z,11Z,14Z))
36	Galactosylhydroxylysine	28-Norcyclomusalenone	
37	Isopimpinellin	Tyrosyl-Serine	
38	3,4-Dihydroxyphenyllactic	Lycoperoside D	

	acid methyl ester	
39	Ursolic acid	Margrapine A

Table S6 Differential metabolites in FGJ during the three different multi-strain fermentation stages

NO	Primary fermentation stage (0-8h)	Post fermentation stage (8-16h)	Final fermentation stage (16-24h)
39	Ursolic acid	Margrapine A	
40	Dihydrofukinolide	Ephedrannin A	
41	Glyceraldehyde	1,3-Diphenyl-1-propanone	
42	4-Hydroxyphenylacetonitrile triacetylrhamnoside	3-Methylthiohexyl hexanoate	
43	Glycyl-L-Leucine	Patuletin 3-(2''-apiosylgentiobioside)	
44	Alpha-dimorphecolic acid	(3a,5b,7a,12a)-24-[(carboxymethyl)amino]-1,12-dihydroxy-24-oxocholan-3-yl-b-D-Glucopyranosiduronic acid	
45	Valyl-Hydroxyproline	Pipericine	
46	Methoxypyrazine	N-[2-(4-Prenyloxyphenyl)ethyl]tiglamide	
47	Malvidin 3-(6-acetylglucoside)	Safflomin C	
48	Isoleucyl-Tryptophan	L-N-(3-Carboxypropyl)glutamine	
49	Mollicellin A	(3R, 6'Z)-3,4-Dihydro-8-hydroxy-3-(6-pentadecenyl)-1H-2-benzopyran-1-one	
50	Dimethyl 3-methoxy-4-oxo-5-(8,11,14-pentadecatrienyl)-2-hexenedioate	Inositol cyclic phosphate	
51	Lycopersiconol	Sagequinone methide A	
52	LysoPC(16:0)		
53	Cholic acid glucuronide		
56	Eremopetasitenin C3		
57	Austalide F		
58	N-2-[4-(3,3-Dimethylallyloxy)phenyl]ethylcinnamide		
59	(Â±)-alpha-Narcotine		
60	Dalbergioidin		

61	Anhydrocinnzeylanine

Table S6 Differential metabolites in FGJ during the three different multi-strain fermentation stages

NO	Primary fermentation stage (0-8h)	Post fermentation stage (8-16h)	Final fermentation stage (16-24h)
62	Thiomorpholine 3-carboxylate		
63	Cervonyl carnitine		
64	(+)-Lyoniresinol 9-glucoside		
65	PE(18:3(6Z,9Z,12Z)/14:0)		
66	Methyl 4,6-di-O-galloyl-beta-D-glucopyranoside		
67	2,3-Dihydroxy-1-(4-hydroxy-3-methoxyphenyl)-1-propanone		
68	PE(14:1(9Z)/14:1(9Z))		
69	5,7-dihydroxy-2-phenyl-6-[3,4,5-trihydroxy-6-(hydroxymethyl)oxan-2-yl]-8-(3,4,5-trihydroxyoxan-2-yl)-4H-chromen-4-one		
70	D-Tagatose 6-phosphate		
71	Sorbitan stearate		
72	Aminofructose 6-phosphate		
73	3-beta-Cellobiosylcellobiose		
74	28-Norcyclomusalenone		
75	2,3-Pentanedione		
76	Ephedrannin A		
77	Sucrose		
78	Chrysoeriol 7-O-(6''-malonyl-glucoside)		
79	(3a,5b,7a,12a)-24-[(carboxymethyl)amino]-1,12-dihydroxy-24-oxocholan-3-yl-b-D-Glucopyranosiduronic acid		
93	Desglucocheirotoxol		
94	D-1-Amino-2-pyrrolidinecarboxylic acid		

95	Epigallocatechin-(4beta->8)-epicatechin 3-O-gallate		

Table S6 Differential metabolites in FGJ during the three different multi-strain fermentation stages

NO	Primary fermentation stage (0-8h)	Post fermentation stage (8-16h)	Final fermentation stage (16-24h)
96	Pandamarilactone 31		
97	Sagequinone methide A		
98	Methyl (9Z)-6'-oxo-6,5'-diapo-6-carotenoate		
99	Diphenyl disulfide		
100	8,11,14-Eicosatrienoic acid		
101	Epigallocatechin-(4beta->8)-catechin		

Table S7 Key differential metabolites in FGJ during the three different multi-strain fermentation stages

Fermentation stages	No	MS2-name	MS2-score	[a]Rt	[b]mz	Mean (0h)	Mean (8h)	Mean (16h)	Mean (24h)	VIP	P-VALUE	Q-VALUE	FOLD-CHANGE
0-8h	1	2',6'-Di-O-acetylononin	0.913	77.370	515.142	0.007	0.048	0.039	0.061	1.745	0.013	0.289	7.385
	2	5,7-dihydroxy-2-phenyl-6-	0.533	314.156	549.158	0.029	0.060	0.045	0.035	1.806	0.033	0.427	2.059
	3	Ephedrannin A	0.467	46.226	557.118	0.232	0.108	0.089	0.097	2.009	0.025	0.381	0.467
	4	Proanthocyanidin A2	0.432	301.176	577.132	0.013	0.006	0.006	0.005	1.967	0.003	0.172	0.469
	5	Epigallocatechin-(4beta->8)-catechin	0.307	260.755	595.142	0.047	0.010	0.010	0.009	2.057	0	0.021	0.213
	6	1-Kestose	0.987	63.783	527.156	0.031	0.212	0.197	0.277	1.793	0.004	0.192	6.894
	7	3-beta-Cellobiosylcellobiose	0.519	48.559	667.225	0.003	0.387	0.395	0.412	1.886	0.003	0.171	132.930
	8	Galactotriose	0.377	47.635	505.175	0.050	0.443	0.404	0.461	2.056	0.0001	0.054	8.732
8-16h	9	Linamarin	0.568	69.763	248.111	0.138	0.123	0.221	0.106	1.858	0.037	1.000	0.480
	10	Dimethyl 3-methoxy-4	0.699	253.828	421.255	0.010	0.024	0.029	0.032	1.996	0.001	0.082	2.321
	11	Palmitoylethanolamide	0.945	623.717	300.289	0.043	0.052	0.020	0.053	1.805	0.026	1.000	0.389
	12	Trifluoromethanesulfonic acid	0.665	26.806	150.968	0.155	0.386	0.100	0.151	2.036	0.001	1.000	0.260
16-24h	13	Tetraacetylethylenediamine	0.899	97.560	229.117	0.150	0.074	0.099	0.202	1.861	0.029	1.000	2.029

[a] RT, retention time; [b] m/z, mass to charge ratio.

Part 3: Analyses of VOCs profiles (Figure S8; Table S8-S10)

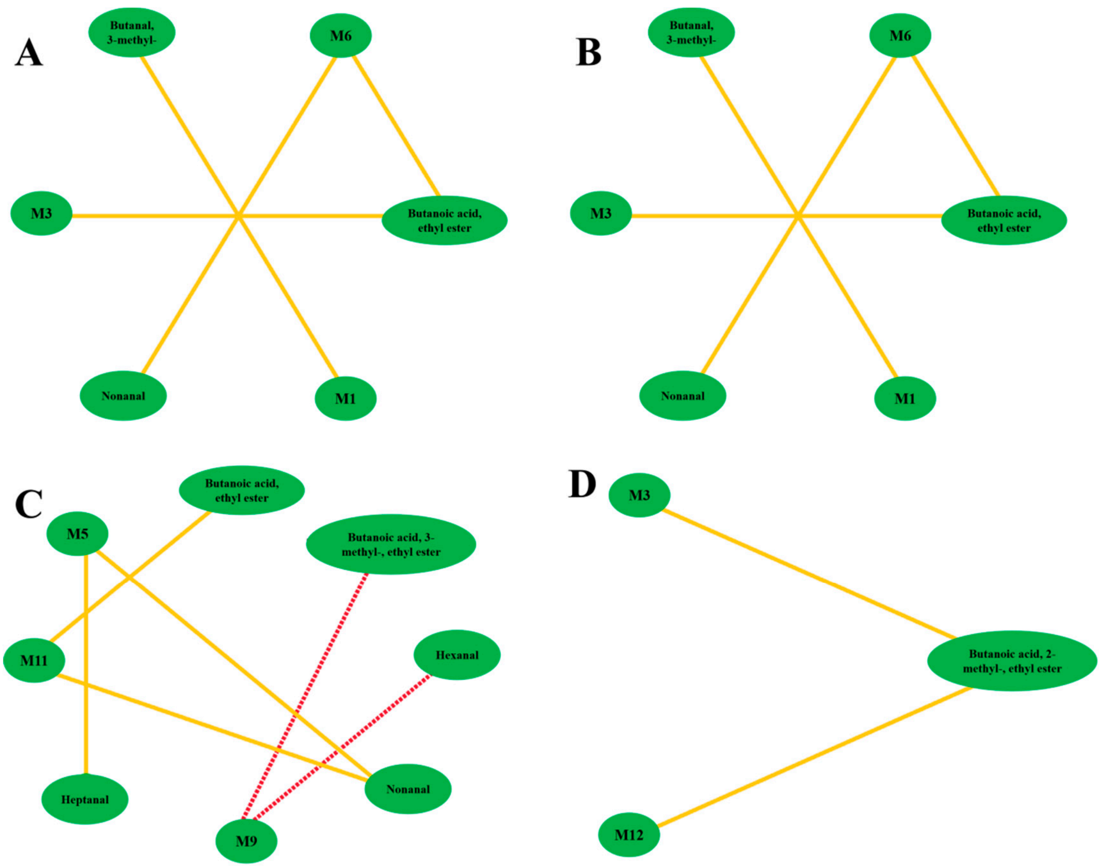

Figure S8 Networks between the VOCs and saccharides in FGJ based on correlation analyses (A: 0h; B: 8h; C: 16h; D: 24h)

Table S8 VOCs in FGJ after 24H of multi-strain fermentation

count	compound	CAS	Formula	mw	Rt
	**Aldehyde**				
1	1-Cyclohexene-1-carboxaldehyde, 2,6,6-trimethyl-	432-25-7	$C_{10}H_{16}O$	119	21.599
2	2-Heptenal, (E)-	18829-55-5	$C_7H_{12}O$	83	13.937
3	2-Hexenal	505-57-7	$C_6H_{10}O$	62	11.084
4	2-Propenal	107-02-8	C_3H_4O	26	2.417
5	3-Hexenal	4440-65-7	$C_6H_{10}O$	69	8.494
6	3-Pentenal, 4-methyl-	5362-50-5	$C_6H_{10}O$	98	3.873
7	Acetaldehyde	75-07-0	C_2H_4O	42	1.732
8	Butanal	123-72-8	C_4H_8O	29	2.694
9	Butanal, 3-methyl-	590-86-3	$C_5H_{10}O$	71	3.193
10	Heptanal	111-71-7	$C_7H_{14}O$	70	9.868
11	Hexanal	66-25-1	$C_6H_{12}O$	56	6.899
12	Methional	3268-49-3	C_4H_8OS	48	17.459
13	Nonanal	124-19-6	$C_9H_{18}O$	98	15.915
14	Octanal	124-13-0	$C_8H_{16}O$	84	12.971
15	Pentanal	110-62-3	$C_5H_{10}O$	58	4.296
16	Propanal, 2-methyl-	78-84-2	C_4H_8O	72	2.189
	**Ester**				
17	2-Hexen-1-ol, acetate, (Z)-	56922-75-9	$C_8H_{14}O_2$	88	14.319
18	2-Propenoic acid, 2-methyl-, ethenyl ester	4245-37-8	$C_6H_8O_2$	41	8.226
19	2,4-Decadienoic acid, ethyl ester, (E,Z)-	3025-30-7	$C_{12}H_{20}O_2$	81	26.645
20	2(3H)-Furanone, 5-butyldihydro-	104-50-7	$C_8H_{14}O_2$	85	28.062
21	2(4H)-Benzofuranone, 5,6,7,7a-tetrahydro-4,4,7a-trimethyl-, (R)-	17092-92-1	$C_{11}H_{16}O_2$	111	35.999
22	3-Hexen-1-ol, acetate, (Z)-	3681-71-8	$C_8H_{14}O_2$	67	13.839
23	Acetic acid ethenyl ester	108-05-4	$C_4H_6O_2$	86	4.284
24	Acetic acid, hexyl ester	142-92-7	$C_8H_{16}O_2$	56	12.572
25	Acetic acid, methoxy-, ethyl ester	3938-96-3	$C_5H_{10}O_3$	61	2.821
26	Acetic acid, methyl ester	79-20-9	$C_3H_6O_2$	74	2.296

Table S8 VOCs in FGJ after 24H of multi-strain fermentation

count	compound	CAS	Formula	mw	Rt
27	Butanoic acid, 2-methyl-, ethyl ester	7452-79-1	$C_7H_{14}O_2$	102	6.185
28	Butanoic acid, 3-hydroxy-, ethyl ester	5405-41-4	$C_6H_{12}O_3$	195	19.254
29	Butanoic acid, 3-methyl-, ethyl ester	108-64-5	$C_7H_{14}O_2$	88	6.609
30	Butanoic acid, ethyl ester	105-54-4	$C_6H_{12}O_2$	71	5.744
31	Butyrolactone	96-48-0	$C_4H_6O_2$	42	21.656
32	Carbamic acid, methylnitroso-, ethyl ester	615-53-2	$C_4H_8N_2O_3$	30	1.567
33	Diethyl carbonate	105-58-8	$C_5H_{10}O_3$	91	7.61
34	Ethyl tiglate	5837-78-5	$C_7H_{12}O_2$	83	11.473
35	Formic acid, ethenyl ester	692-45-5	$C_3H_4O_2$	29	2.409
36	Hexanoic acid, 3-hydroxy-, ethyl ester	2305-25-1	$C_8H_{16}O_3$	46	23.113
37	Hexanoic acid, 5-oxo-, ethyl ester	13984-57-1	$C_8H_{14}O_3$	108	24.115
38	Hexanoic acid, ethyl ester	123-66-0	$C_8H_{16}O_2$	88	11.420
39	Isobutyl acetate	110-19-0	$C_6H_{12}O_2$	43	6.704
40	Pentanoic acid, ethyl ester	539-82-2	$C_7H_{14}O_2$	88	8.409
41	Propanoic acid, 2-oxo-, ethyl ester	617-35-6	$C_5H_8O_3$	43	12.455
42	Propanoic acid, ethyl ester	105-37-3	$C_5H_{10}O_2$	57	3.901
	**Ketone**				
43	1-Penten-3-one	1629-58-9	C_5H_8O	55	5.257
44	1H-Pyrrole-2,5-dione, 3-ethyl-4-methyl-	20189-42-8	$C_7H_9NO_2$	67	34.802
45	2-Heptanone, 6-methyl-	928-68-7	$C_8H_{16}O$	71	21.181
46	2-Octanone	111-13-7	$C_8H_{16}O$	58	12.893
47	2-Pentadecanone, 6,10,14-trimethyl-	502-69-2	$C_{18}H_{36}O$	137	32.296
48	2,3-Hexanedione	3848-24-6	$C_6H_{10}O_2$	43	8.312
50	2(5H)-Furanone	497-23-4	$C_4H_4O_2$	84	24.528
51	3-Chloro-2,4-pentanedione	1694-29-7	$C_5H_7ClO_2$	119	12.546
52	3-Hexanone, 4-methyl-	17042-16-9	$C_7H_{14}O$	85	19.708
53	5-Hepten-2-one, 6-methyl-	110-93-0	$C_8H_{14}O$	111	14.384
54	Acetoin	513-86-0	$C_4H_8O_2$	45	12.917

Table S8 VOCs in FGJ after 24H of multi-strain fermentation

count	compound	CAS	Formula	mw	Rt
55	Acetone	67-64-1	C_3H_6O	58	2.207
56	Cyclononanone	3350-30-9	$C_9H_{16}O$	55	19.569
	**Acid**				
57	Acetic acid	64-19-7	$C_2H_4O_2$	60	17.418
	**Alcohol**				
58	1-Butanol	71-36-3	$C_4H_{10}O$	56	9.176
59	1-Heptanol, 4-methyl-	817-91-4	$C_8H_{18}O$	41	20.456
60	1-Hexen-3-ol	4798-44-1	$C_6H_{12}O$	57	12.161
61	1-Nonanol	143-08-8	$C_9H_{20}O$	56	22.882
62	1-Octen-3-ol	3391-86-4	$C_8H_{16}O$	72	17.615
63	1-Penten-3-ol	616-25-1	$C_5H_{10}O$	57	9.557
64	2-Chloroethanol	107-07-3	C_2H_5ClO	44	3.519
65	2-Hexen-1-ol, (E)-	928-95-0	$C_6H_{12}O$	67	16.494
66	3-Buten-1-ol, 3-methyl-	763-32-6	$C_5H_{10}O$	67	12.053
67	3-Hexen-1-ol, (E)-	928-97-2	$C_6H_{12}O$	74	15.881
68	4-Methyl-2-hexanol	2313-61-3	$C_7H_{16}O$	71	14.157
69	Ethanol	64-17-5	C_2H_6O	46	3.550
70	Methyl Alcohol	67-56-1	CH_4O	31	2.961
	**Other**				
71	1-Penten-3-one, 2-methyl-	25044-01-3	$C_6H_{10}O$	69	6.474
72	1,4-Oxathiane	15980-15-1	C_4H_8OS	104	15.021
73	2-Cyclohexen-1-ol, 2,6,6-trimethyl-	54345-59-4	$C_9H_{16}O$	125	31.210
74	2,5-Furandione, 3,4-dimethyl-	766-39-2	$C_6H_6O_3$	54	24.022
75	Acetamide	60-35-5	C_2H_5NO	59	24.910
76	Acetic anhydride	108-24-7	$C_4H_6O_3$	43	2.821
77	Cyclobutane, methylene-	1120-56-5	C_5H_8	52	1.628
78	Dodecane	112-40-3	$C_{12}H_{26}$	85	10.238
79	Hexanenitrile	628-73-9	$C_6H_{11}N$	54	13.188

Table S8 VOCs in FGJ after 24H of multi-strain fermentation

count	compound	CAS	Formula	mw	Rt
80	Isopropylcyclobutane	872-56-0	C_7H_{14}	55	2.448
81	Pinocarvone	30460-92-5	$C_{10}H_{14}O$	108	20.320
82	Pyrazine, tetramethyl-	1124-11-4	$C_8H_{12}N_2$	136	18.169
83	Pyrazine, trimethyl-	14667-55-1	$C_7H_{10}N_2$	122	16.291
84	Succinimide	123-56-8	$C_4H_5NO_2$	99	38.172
85	Tridecane	629-50-5	$C_{13}H_{28}$	57	13.337

Table S9 Carbohydrates (mean relative abundance > 0.5%) in FGJ during multi-strain fermentation stages

No	Metabolites	[a]RT(min)	[b]m/z	Mean (0h)	Mean (8h)	Mean (16h)	Mean (24h)	Class
M1	L-Gulose	67.158	203.051	15.296	6.805	14.354	18.427	Carbohydrates and carbohydrate conjugates
M2	Phenylethyl primeveroside	281.914	439.155	0.847	0.866	0.836	0.608	Carbohydrates and carbohydrate conjugates
M3	2-O-Acetylarbutin	217.317	315.104	1.395	1.404	1.367	1.300	Carbohydrates and carbohydrate conjugates
M4	Hydroxytyrosol	249.069	317.119	11.169	11.130	10.886	11.002	Amino acids, peptides, and analogues
M5	1-O-glucoside	291.569	477.192	1.161	1.179	1.163	1.181	Carbohydrates and carbohydrate conjugates
M6	Kanokoside A	277.363	393.115	0.619	0.608	0.603	0.609	Carbohydrates and carbohydrate conjugates
M7	Garcimangosone D	293.056	343.172	1.523	1.305	1.711	0.902	Carbohydrates and carbohydrate conjugates
M8	Jasmolone glucoside	214.414	180.086	7.531	7.397	7.793	7.684	Carbohydrates and carbohydrate conjugates
M9	Glucosamine	458.434	180.086	1.501	1.407	1.411	1.501	Carbohydrates and carbohydrate conjugates
M10	L-Galactose	136.806	198.096	44.125	48.468	51.924	48.579	Carbohydrates and carbohydrate conjugates
M11	Glucosylisomaltol	47.381	289.091	0.717	0.644	0.586	0.593	Carbohydrates and carbohydrate conjugates
M12	Ethyleta-D-glucopyranoside	101.955	209.101	4.434	4.350	4.291	4.312	Carbohydrates and carbohydrate conjugates

[a] RT, retention time; [b]m/z, mass to charge ratio.

Table S10 Carboxylic acids and derivatives, and fatty acyls (mean relative abundance > 0.5%) in FGJ during multi-strain fermentation stages

No	Metabolites	[a]RT(min)	[b]m/z	Mean (0h)	Mean (8h)	Mean (16h)	Mean (24h)	Class
M1	Diethyl fumarate	187.770	173.078	4.698	4.675	4.388	4.818	Fatty Acyls
M2	Betaine	781.100	118.086	5.702	5.919	5.883	5.904	Carboxylic acids and derivatives
M3	Bisnorbiotin	71.540	217.067	5.334	6.158	5.798	5.456	Organic acids and derivatives
M4	D-Proline	47.620	116.070	36.555	34.105	34.187	33.175	Carboxylic acids and derivatives
M5	Kanokoside A	291.560	477.192	1.161	1.179	1.163	1.181	Carboxylic acids and derivatives
M6	3,3,5-triiodo-L-thyronine-	88.160	132.101	21.848	22.095	21.062	22.312	Carboxylic acids and derivatives
M7	6-Aminopenicillanic acid	86.730	217.067	1.377	1.379	1.346	1.412	Carboxylic acids and derivatives
M8	L-Histidine	42.330	156.076	2.704	2.681	2.557	2.660	Carboxylic acids and derivatives
M9	1-deoxy-1-(N6-lysino)-D-	39.680	134.044	1.875	1.396	1.299	1.218	Carboxylic acids and derivatives
M10	Lauroyl diethanolamide	494.630	288.252	5.971	6.020	6.070	6.290	Fatty Acyls
M11	(alpha-D-mannosyl)7-beta-	43.620	90.055	7.669	7.122	7.177	6.980	Carboxylic acids and derivatives
M12	2-Methyl-3-hydroxyvaleric	249.040	133.086	1.574	1.559	1.506	1.488	Fatty Acyls
M13	L-Glutamic acid	40.560	148.060	5.577	4.297	3.644	3.520	Carboxylic acids and derivatives
M14	Kojibiose	46.980	325.112	7.882	9.383	9.324	8.996	Fatty Acyls
M15	N-Acryloylglycine	69.110	130.050	2.056	1.962	2.225	1.965	Carboxylic acids and derivatives
M16	L-Allothreonine	43.780	120.065	4.112	3.884	3.924	3.917	Organic acids and derivatives
M17	L-Methionine	55.430	150.058	2.624	2.744	2.670	2.719	Organic acids and derivatives
M18	N2-Fructopyranosylarginine	44.230	337.170	1.286	1.524	1.646	1.563	Carboxylic acids and derivatives
M19	(2E,4E)-2,7-Dimethyl-2,4-o	165.380	199.100	1.535	1.494	1.743	1.420	Fatty Acyls
M20	Argininosuccinic acid	48.780	291.129	5.165	5.072	4.673	4.907	Carboxylic acids and derivatives
M21	Dihydroceramide	663.810	330.299	1.506	1.514	1.493	1.552	Carboxylic acids and derivatives
M22	Vinylacetylglycine	793.910	144.065	3.097	3.551	3.031	3.27	Carboxylic acids and derivatives
M23	Docosanamide	674.180	340.356	4.009	2.092	5.347	4.619	Fatty Acyls
M24	Histidinyl-Asparagine	44.270	270.117	1.823	1.759	1.760	1.791	Carboxylic acids and derivatives
M25	Corchoionol C 9-glucoside	485.150	387.191	1.323	1.260	1.245	1.208	Fatty Acyls
M26	Palmitic acid	477.190	274.273	50.276	44.012	48.891	48.097	Fatty Acyls
M27	2,4,12-Octadecatrienoic	675.110	334.310	2.582	2.333	2.365	2.572	Fatty Acyls

[a] RT, retention time; [b]m/z, mass to charge ratio.
